# Artificial Intelligence Applied to Chest X-ray: A Reliable Tool to Assess the Differential Diagnosis of Lung Pneumonia in the Emergency Department

**DOI:** 10.3390/diseases11040171

**Published:** 2023-11-20

**Authors:** Davide Ippolito, Cesare Maino, Davide Gandola, Paolo Niccolò Franco, Radu Miron, Vlad Barbu, Marco Bologna, Rocco Corso, Mihaela Elena Breaban

**Affiliations:** 1 Department of Diagnostic Radiology, Fondazione IRCCS San Gerardo dei Tintori, Via Pergolesi 33, 20900 Monza, Italy; davide.atena@tiscalinet.it (D.I.); gandolad@gmail.com (D.G.); francopaoloniccolo@gmail.com (P.N.F.); rocco.corso@irccs-sangerardo.it (R.C.); 2 School of Medicine, University of Milano-Bicocca, Via Cadore 48, 20900 Monza, Italy; 3 Sentic Lab, Strada Elena Doamna 20, 700398 Iași, Romania; radu.miron@senticlab.onmicrosoft.com (R.M.); vlad.barbu@senticlab.onmicrosoft.com (V.B.); 4 Synbrain, Corso Milano 23, 20900 Milan, Italy; marco.bologna@synbrain.ai; 5 Faculty of Computer Science, “Alexandru Ioan Cuza” University of Iasi, Strada General Henri Mathias Berthelot 16, 700483 Iași, Romania

**Keywords:** artificial intelligence, chest X-ray, SARS-CoV-2, COVID-19

## Abstract

Background: Considering the large number of patients with pulmonary symptoms admitted to the emergency department daily, it is essential to diagnose them correctly. It is necessary to quickly solve the differential diagnosis between COVID-19 and typical bacterial pneumonia to address them with the best management possible. In this setting, an artificial intelligence (AI) system can help radiologists detect pneumonia more quickly. Methods: We aimed to test the diagnostic performance of an AI system in detecting COVID-19 pneumonia and typical bacterial pneumonia in patients who underwent a chest X-ray (CXR) and were admitted to the emergency department. The final dataset was composed of three sub-datasets: the first included all patients positive for COVID-19 pneumonia (n = 1140, namely “COVID-19+”), the second one included all patients with typical bacterial pneumonia (n = 500, “pneumonia+”), and the third one was composed of healthy subjects (n = 1000). Two radiologists were blinded to demographic, clinical, and laboratory data. The developed AI system was used to evaluate all CXRs randomly and was asked to classify them into three classes. Cohen’s κ was used for interrater reliability analysis. The AI system’s diagnostic accuracy was evaluated using a confusion matrix, and 95%CIs were reported as appropriate. Results: The interrater reliability analysis between the most experienced radiologist and the AI system reported an almost perfect agreement for COVID-19+ (κ = 0.822) and pneumonia+ (κ = 0.913). We found 96% sensitivity (95% CIs = 94.9–96.9) and 79.8% specificity (76.4–82.9) for the radiologist and 94.7% sensitivity (93.4–95.8) and 80.2% specificity (76.9–83.2) for the AI system in the detection of COVID-19+. Moreover, we found 97.9% sensitivity (98–99.3) and 88% specificity (83.5–91.7) for the radiologist and 97.5% sensitivity (96.5–98.3) and 83.9% specificity (79–87.9) for the AI system in the detection of pneumonia+ patients. Finally, the AI system reached an accuracy of 93.8%, with a misclassification rate of 6.2% and weighted-F1 of 93.8% in detecting COVID+, pneumonia+, and healthy subjects. Conclusions: The AI system demonstrated excellent diagnostic performance in identifying COVID-19 and typical bacterial pneumonia in CXRs acquired in the emergency setting.

## 1. Introduction

During previous decades, a broader and more progressive application of artificial intelligence (AI) in the biomedical field was registered, particularly in radiology, thanks to the more structured and robust use of machine learning systems [1,2]. In clinical practice, AI can help radiologists determine the presence of a specific pathological process, especially in the emergency setting, where a single person often handles pressure and a high amount of data. Evaluating chest X-rays (CXRs) in patients admitted to the emergency department is one of the most critical scenarios which radiologists should face. This aspect was especially true during the COVID-19 pandemic waves [3].

In this setting, AI models have been developed to assist medical practitioners in detecting and diagnosing COVID-19. Machine learning algorithms are used to fit models on medical imaging data [such as CXRs and computed tomography (CT)] to help identify patterns associated with the virus. These AI tools can help healthcare professionals make quicker and more accurate diagnoses during waves of pandemic [4]. Moreover, the application of AI can help reduce human bias in the diagnosis of COVID-19, as reported by Bercean et al. [5]. The authors revealed that radiologists overestimated the percentage of lung involvement by 10.23 ± 4.65% and 15.8 ± 6.6%, respectively, while with the AI support, the absolute overestimation error was reduced.

It has been widely confirmed that AI-assisted diagnosis is a valuable screening tool that might shorten patient waiting times, simplify the workflow, reduce the workload of radiologists, and enable radiologists to respond more quickly and effectively in the emergency setting [6].

Nowadays, most current studies use CXRs or CTs to classify COVID-19 pneumonia and other forms of pneumonia [7]. In a recent paper, Bouchareb et al. [8] deeply revised the importance of AI as an assistive tool in diagnosing and prognosis of COVID-19 patients, especially by applying deep learning (DL) approaches. AI techniques used for CXR allow for identifying the most common and typical findings, namely ground-glass opacities (GGOs) and consolidations, especially with a bilateral, lower, and peripheral zone distribution [9]. The authors, citing the work by Bukhari et al. [10], underlined the usefulness of ResNet-50 CNN architectures in identifying patients with COVID-19 pneumonia, typical bacterial pneumonia, and with normal CXR, with an overall diagnostic accuracy of 92.8%. Similar results were reported by Apostolopoulos et al. [11], who used different CNN models for differential diagnosis between COVID-19 pneumonia and other pathological findings such as pulmonary edema, pleural effusion, and chronic obstructive pulmonary disease. The authors found a 99.2% accuracy in the diagnosis of COVID-19 pneumonia.

Even if CXRs have plenty of potential, such as in determining both triage and consequent management [12], different practice issues should be mentioned: image quality, reader’s experience, patient’s habitus, and lung inflation are the most typical bacterial pitfalls in everyday practice [13]. In this setting, AI can help radiologists acquire diagnostic confidence in evaluating CXR in patients suspected of COVID-19 pneumonia [7,14,15].

A significant concern addressed in several studies is the robustness of the AI models for classification [9,10,11], focusing on DL models that lack interpretation. Classification models, even those with a good performance, may not be enough to analyze a CXR properly. Their good performance may be caused by bias inferred from the image’s background, so actual diagnosis in newer scenarios may be misleading. In this regard, a previously published study [16] replicated the modeling choices of high-performance models for COVID-19 classification found in the literature and experimented with data from the GitHub-COVID repository. Its main findings show that these models “fail to learn the true underlying pathology and instead leverage spurious associations between presence or absence of COVID-19 and radiographic features that reflect variations in image acquisition”. These studies usually provide visualizations of the affected areas of the lungs by using salient detection or grad-cam as techniques to highlight the affected areas. Still, those techniques may fail to identify the affected area(s). Multi-tasking models that classify and segment with inference may be preferable to avoid this issue. An example of this model was reported by Li et al. [17]. However, this model is based on CT imaging, an unsuitable imaging technique for all patients in the emergency department. To our knowledge, no similar models exist for CXRs.

On these bases, this study aims to test the diagnostic performance of an AI system in detecting COVID-19 lung involvement in patients who underwent CXR in the emergency department. The secondary aim was to test the AI system in promptly identifying typical bacterial pneumonia.

## 2. Materials and Methods

This study was performed under the Declaration of Helsinki. The institutional review board stated that the approval was not necessary due to the retrospective and anonymous nature of the present study, following article 89 of the GDPR EU Regulation 2016/679. Consent was verbally obtained from participants; however, due to the pandemic, they were not required to sign it. This was a retrospective observational analysis based on previously collected routine care data. All radiological and clinical data were anonymized before being analyzed.

### 2.1. Patients and CXRs Datasets

All patients who underwent bedside CXR obtained in the anteroposterior projection through a portable X-ray machine (Dx-D 100—Agfa Healthcare—with fixed setting regarding kV and mAs) in the emergency department were retrospectively collected by a radiologist resident with four years of experience, who created three different subdatasets according to the period of acquisition. The radiologist resident collected all CXRs acquired in the specific periods reported below. Based on his experience, CXRs with motion artifacts or technically inadequate were excluded at this time point.

The sub dataset 1 included all CXRs acquired between 1 April and 1 October 2020. During this period, all patients checking into the emergency department of a tertiary care referral center (San Gerardo Hospital—Monza–Italy) suspected of COVID-19 infection underwent CXR at admission. The inclusion criteria for dataset 1 were: (1) positive reverse transcription-polymerase chain reaction (RT-PCR) test, and (2) CXR performed with an interval time not higher than 24 h from the RT-PCR test. The first dataset was initially composed of 6320 CXRs, in particular, 1010 (15.9%) acquired in April 1004 (15.9%) in May, 1113 (17.6%) in June, 979 (15.5%) in July, 1022 (16.2%) in August, and 1192 (18.9%) in September. Considering the large amount of data to be read by the two radiologists, we decided to select 190 CXRs per month randomly. The randomization process was made by using a freeware webpage (random.org).

Sub dataset 2 comprised all CXRs acquired before the COVID pandemic until September 2018 and included patients checked into the emergency department for suspicion of pulmonary infection. All patients underwent laboratory tests, including white blood cell count (WBC), red blood cell count (RBC), C-reactive protein (CRP) level, and symptoms in line with pulmonary infection, including fever and cough. The final diagnosis was confirmed with a follow-up radiological examination (including serial CXRs and chest CT) or laboratory data (i.e., Legionella or Pneumococcal urinary antigen).

Sub dataset 3 was composed of CXRs acquired before the COVID pandemic until September 2018 and included all patients checked into the emergency department for other than pulmonary infection (e.g., trauma, chest pain, or pneumothorax). All these CXRs were confirmed as negative (healthy subjects) according to the discharge of patients or the evaluation of follow-up examinations (e.g., CXRs or CT).

**Figure 1:**
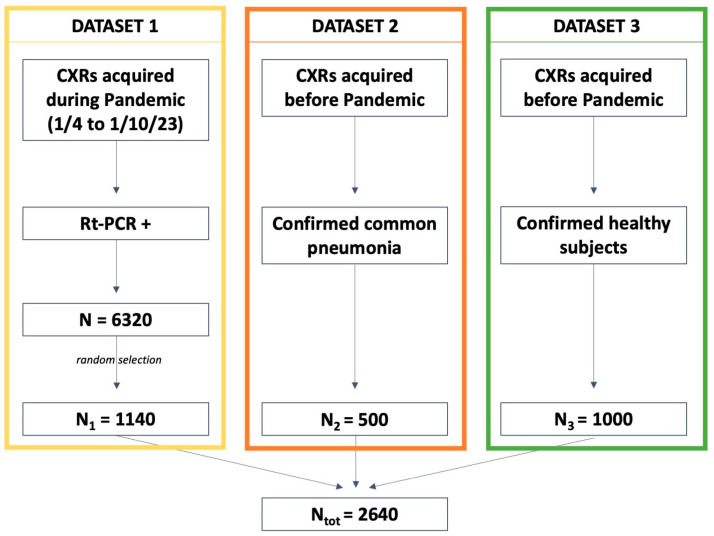
Details regarding the datasets. RT-PCR: reverse transcriptase-polymerase chain reaction.

As reported in Figure 1, the final dataset was composed of 2640 CXRs, of whom 1140 (43.2%), 500 (18.9%), and 1000 (37.9%) belonged to sub-datasets 1, 2, and 3, respectively.

### 2.2. Radiologists’ Evaluation

Two radiologists with 10 and 15 years of experience in emergency and lung imaging evaluated all CXRs belonging to the final dataset, blinded to demographical, clinical, and laboratory data. To avoid bias, the radiologists were also blinded to the date of CXR acquisition. For each patient, they were asked to determine the presence of pathological features, dividing them into COVID-19 positive (COVID-19+), positive for typical lung pneumonia (pneumonia+), and healthy. Finally, to avoid recalling bias, the CXRs were evaluated in a random order.

### 2.3. AI System: Technical Details and Image Analysis

We developed a multi-tasking model that could perform both image-level classification (as COVID-19, COVID-19 negative, or healthy) and object detection/instance segmentation (for consolidations and interstitial patterns).

**Figure 2:**
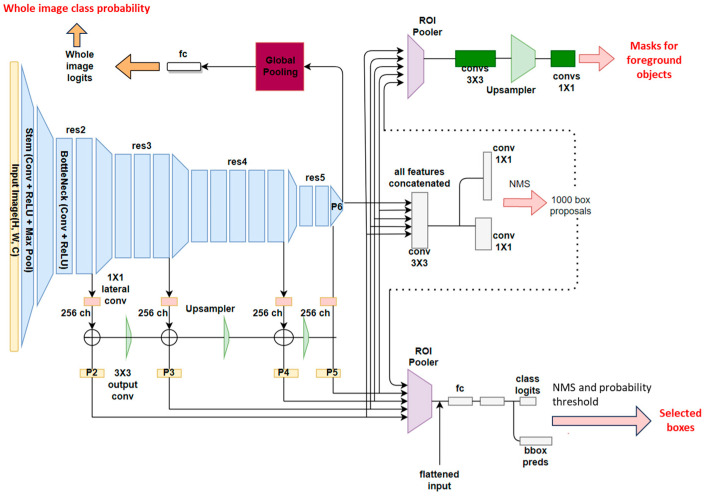
Architectural scheme of the modified, multi-tasking Mask R-CNN model used for the study.

Mask R-CNN [18] was the base architecture used in the study. To develop a neural network that performed semantic segmentation and classification on CXRs, a modified, multi-tasking Mask R-CNN was used starting from an implementation that can be found on detectron2 [@misc{wu2019detectron2, author = {Yuxin Wu and Alexander Kirillov and Francisco Massa and Wan-Yen Lo and Ross Girshick}, title = {Detectron2}, method of publication = {\url{https://github.com/facebookresearch/detectron2}}, year = {2019}}], which is the mask_rcnn_R_50_FPN_3x (https://github.com/facebookresearch/detectron2 was accessed on 31 July 2022). A schema representing the leading architecture of the network and the input and outputs is illustrated in Figure 2.

A multi-tasking Mask R-CNN, such as the one that has been used in this study, is made up of the following blocks: (1) a backbone network, (2) a region proposal network (RPN), (3) a bounding box (or ROI) head, and (4) a classification head.

The backbone is common to all the tasks that the neural network performs. The purpose of this network is to extract feature maps from the input image. These features are not user-defined but instead are automatically learned during training and represent some inner information inside the image, visible at different scales. The convolutional backbone is made by a ResNet50, which is made by a succession of convolutional and max pooling layers [19] inserted into a feature pyramid network [20]. Different layers of the networks work at different resolutions (res2, res3, etc.), with feature maps from the latest layers having lower resolution but higher receptive fields (a portion of the original image used to compute the information in a particular pixel of the map). The latest layer of the network contains the tensor, which captures the most high-level information (called P6 as the top layer of the pyramid); however, intermediate feature maps are also extracted as typical of the feature pyramid network. At the end of each resolution level, a 1 × 1 convolution layer allows extracting 256 feature maps with the exact resolution of the corresponding res layer. P2 indicates the tensors extracted by the feature pyramid networks in the different levels to P5, with lower levels representing lower pyramids with lower receptive fields and lower-level information. Image classification uses information only from P6 (as for the original ResNet50 developed for classification). At the same time, the RPN also considers the local information provided by P2–P5 (in addition to the one of P6).

The RPN [21] predicts, given the output of the FPN (concatenated tensors P2 to P6), the indicated objectness scores and regression scores on the proposed anchors. Here, the network learns to detect objects of interest and background objects. After the predictions, NMS (non-maximal-suppression) keeps non-overlapping bounding boxes. The output of the RPN is then sent to the heads that produce the final prediction of bounding boxes and segmentation.

The region of interest (ROI) box heads module uses the boxes detected in the RPN module and the features extracted from the backbone. Using an ROI pooler [18], we put together each box found in the RPN module and its corresponding parts from tensors P2, P3, P4, or P5. Given these, linear layers extract features for classifying the object contours and bounding box regression. The types of objects identified by the network of our interest are consolidation and interstitial patterns.

The classification head was added to the architecture to output scores (logits) for the negative classes of COVID-19, healthy, and COVID-19. It used global average pooling and linear layers to extract the probabilities for the three output classes. The model uses the P6 features extracted by the backbone to make the classification.

The training was performed for 100,000 steps, with a batch size of 2, using stochastic gradient descent. We clipped gradients above one and used a warm-up multi-step learning rate policy with an initial value of the learning rate of 0.000125. We used a linear warm-up for 1000 steps and a weight decay of 1 × 10^−4^. The final loss was the sum of losses from the RPN, Fast-RCNN detector, and classification head. As preprocessing steps, the input DICOM files were converted to PNG, followed by histogram equalization. As augmentation steps, we used random resizing, with the smallest size from the set of [640, 672, 704, 736, 768, 800, 1333], Gaussian blur, and Gaussian noise. All the parameters and augmentation steps were chosen heuristically based on the results of preliminary analyses. The output of the new Mask R-CNN was a tuple containing boxes, masks, and output classes for each object detected in the image (Figure 2). No early stopping was used, but the latest model was considered since we considered that using a pre-trained model and the availability of a large image dataset was enough to avoid overfitting the model.

The radiologist resident analyzed the final dataset using the newly developed system (AID Chest XR—M.S. HUMANAID, EmmeEsse srl—Milan, Italy). CXRs were also evaluated in a random order in this setting to avoid recall bias.

### 2.4. Statistical Analysis

We first evaluated the agreement between radiologists and then used data labels from the most experienced one for further analysis. The agreement between the most experienced radiologist and the AI system was assessed using the Cohen Kappa coefficient (0.00–0.20 indicates slight agreement; 0.21–0.40, fair agreement; 0.41–0.60, moderate agreement; 0.61–0.80, substantial agreement; and 0.81–1.00, almost perfect agreement).

To evaluate specific diagnostic values for COVID-19 pneumonia and typical bacterial pneumonia, we tested the most experienced radiologist and the AI system with a first mixed dataset composed of sub-datasets 1 and 3 (n = 2140) and sub-datasets 2 and 3 (n = 1500), respectively. For computing these values, we collected true positives (TPs), true negatives (TNs), false positives (FPs), and false negatives (FNs). Based on them we computed sensitivity [=TPs/(TPs + FNs)], specificity = TNs/(TNs + FP)s], positive predictive value (PPV) [=TPs/(TPs + FPs)], negative predictive value (NPV) [=TNs/(TNs + FNs)], and balanced accuracy [=(sensitivity + specificity)/2] by using a 2 × 2 confusion matrix. The 95% confidence intervals (95% CIs) were reported as appropriate.

We performed a second analysis on the final dataset to test the ability of the AI system to discriminate between COVID-19 pneumonia, typical bacterial pneumonia, and healthy patients. We computed a 3 × 3 confusion matrix to collect its diagnostic values. The precision, recall, F−1 score for each class, accuracy, misclassification rate, and weighted F1 were reported as appropriate.

All the statistical analyses were performed using IBM SPSS 26.0 (SPSS Incorporated, Chicago, IL, USA).

## 3. Results

### 3.1. Reliability Analysis

The agreement between the two radiologists was almost perfect in identifying COVID-19+ (κ = 0.812) and pneumonia+ (κ = 0.901) patients.

Similarly, the interrater reliability analysis between the most experienced radiologist and the AI system reported an almost perfect agreement for COVID-19+ (κ = 0.822) and pneumonia+ (κ = 0.913).

### 3.2. COVID-19 Pneumonia

To test the most experienced radiologist and the AI system regarding COVID-19 pneumonia, we used CXRs belonging to datasets 1 and 3 (n = 2140).

**Figure 3:**
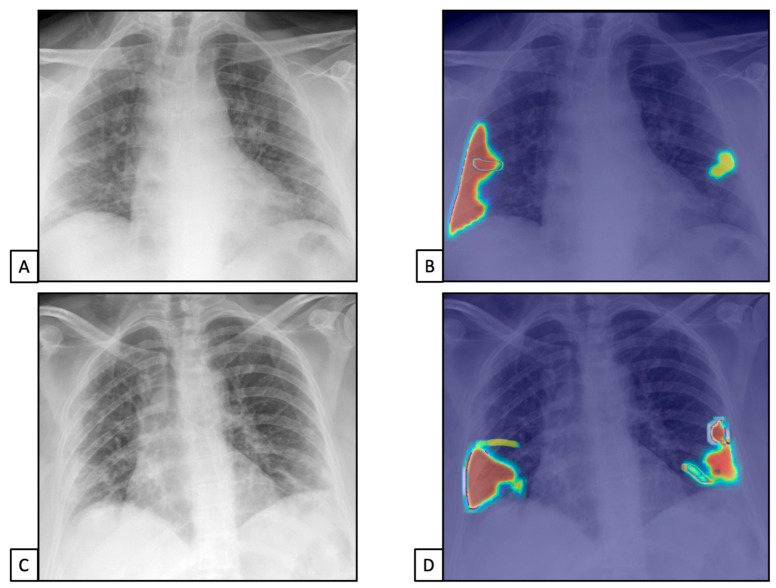
Chest X-rays of patients admitted to the emergency department with the suspicion of COVID-19 infection belonged to dataset 1. (**A**,**C**) represent CXRs acquired at the bedside, showing multiple slight interstitial and alveolar opacities located peripherally and with a lower distribution. (**B**,**D**) The AI system analysis obtained in a few seconds displays the pathological zones. The AI system reported a high suspicion of COVID-19 infection (99.99%). The final diagnosis was lung involvement by COVID-19 pneumonia.

The TPs and TNs, according to the radiologist, were similar to the AI system ones [n = 1450 (67.8) and n = 503 (23.5), and n = 1419 (66.3) and n = 515 (24.1), respectively]. Similar results were found for FPs and FNs [n = 127 (5.9), n = 60 (2.8), n = 127 (5.9), and n = 79 (3.7), respectively]. This resulted in 96% sensitivity (95%CIs = 94.9–96.9) and 79.8% specificity (76.4–82.9) for the radiologist and 94.7% sensitivity (93.4–95.8) and 80.2% specificity (76.9–83.2) for the AI system (Figure 3). The radiologist showed 91.9% PPV (90.7–93.0) and 89.3% NPV (86.7–91.5), while the AI system showed 91.7% PPV (90.5–92.8) and 86.7% NPV (83.9–89.0).

Finally, the accuracy was similar between the radiologist and the AI system [91.2% (89.9–92.4) vs. 90.3% (89.0–91.6), respectively].

All data are summarized in Table 1 and Table 2.

### 3.3. Typical Bacterial Pneumonia

To test the most experienced radiologist and the AI system regarding COVID-19 pneumonia, we used CXRs belonging to datasets 2 and 3 (n = 1500).

**Figure 4:**
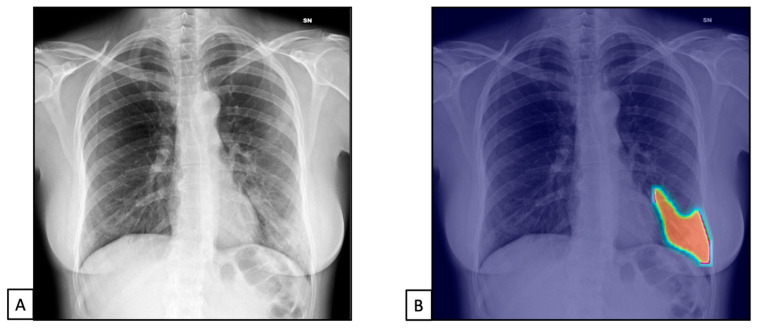
Chest X-rays of patients admitted to the emergency department with respiratory distress and fever belonged to dataset 2. (**A**) represents CXR acquired at the bedside, showing compact typical opacities in the left lung lower zones. (**B**) represents AI system analysis, showing the pathological zones. The AI system reported a high suspicion of pneumonia, not typical for COVID-19 (99.99%). The final diagnosis was lung pneumonia due to Streptococcus pneumoniae.

**Figure 5:**
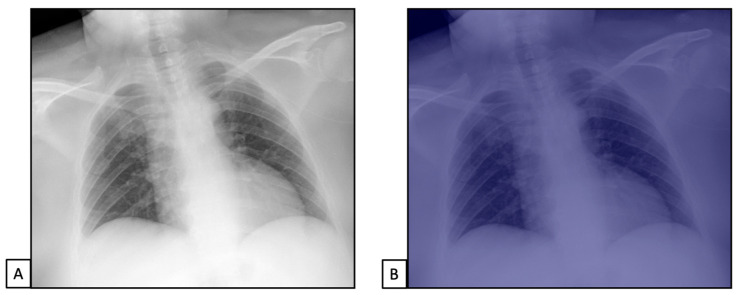
Chest X-rays of patients admitted to the emergency department with thoracic pain belonged to dataset 3. (**A**) represents CXR acquired at the bedside, showing no pathological findings. (**B**) represents AI system analysis, showing no pathological zones. The AI system reported a high suspicion of healthy subjects (98.5%). The final diagnosis was thoracic pain due to myocardial infarction.

The TPs and TNs, according to the radiologist, were similar to the AI system ones [n = 1218 (81.2) and n = 235 (15.6), and n = 1191 (79.4) and n = 234 (15.6), respectively], both with similar FPs and FNs [n= 127 (5.9) for both, and n = 60 (2.8) and n = 79 (3.7), respectively]. This resulted in 97.9% sensitivity (98.0–99.3) and 88% specificity (83.5–91.7) for the radiologist and 97.5% sensitivity (96.5–98.3) and 83.9% specificity (79.0–87.9) for the AI system (Figure 4 and Figure 5).

The radiologist showed 97.4% PPV (96.5–98.1) and 94% NPV (90.4–96.3), while the AI system showed 96.3% PPV (95.2–97.1) and 88.6% NPV (84.5–91.7).

Finally, the accuracy was similar between the radiologist and the AI system [96.9% (95.8–97.7) vs. 95% (93.7–96), respectively].

All data are summarized in Table 1 and Table 2.

### 3.4. Diagnostic Accuracy of AI System

The confusion matrix showed a precision of 97.1% for COVID+, with a recall of 90.1% and a f1-score of 93.7%. Analogous results were found for pneumonia+ patients with 94.8% precision, 95.4% recall, and 95.1% f1-score. Also, in cases of healthy subjects, we found 90.1% precision, 96.8% recall, and 93.3% f1-score. Finally, the AI system reached an accuracy of 93.8%, with a misclassification rate of 6.2% and 93.8% weighted-F1.

**Figure 6:**
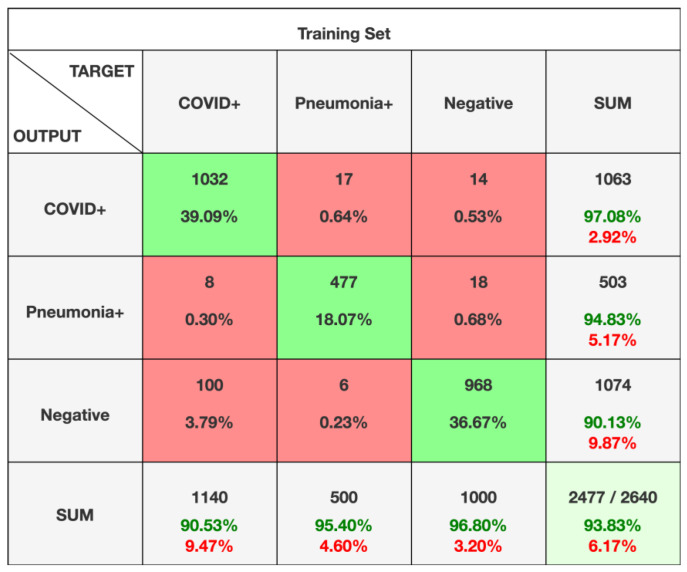
Confusion matrix for the AI system. Data included in the green squares represent correctly labeled CXRs. Data contained in the red squares represent incorrectly labelled CXRs.

The confusion matrix is reported in Figure 6.

## 4. Discussion

Our study demonstrates that an AI-based system can be considered a reliable tool in promptly identifying lung abnormalities, particularly helping the differential diagnosis between COVID-19 pneumonia and typical bacterial pneumonia. In the present study, we aimed to test the AI system using 2640 CXRs acquired during different time points before and during the pandemic. Before trying it, we decided to evaluate the interrater reliability analysis between two expert radiologists in lung and emergency radiology: our results show that the agreement between them was almost perfect for all the datasets evaluated (κ = 0.812–0.980). We then decided to compare the interrater reliability between human readers and the AI-based system, demonstrating that the agreement between them was almost perfect regarding COVID-19 pneumonia (κ = 0.822), typical bacterial pneumonia (κ = 0.913), and healthy subjects (κ = 0.970). These results stress that the agreement increased for usual bacterial pneumonia due to the more evident radiological findings. When comparing the diagnostic performance of human readers and an AI-based system in the diagnosis of COVID-19 pneumonia, we found that both reported high sensitivity (96% and 94.7%, respectively) and specificity values (79.8% and 80.2%, respectively), with reasonable accuracy (91.2% and 90.3%, respectively). The same results were found regarding typical bacterial pneumonia: the radiologists’ sensitivity and specificity rose to 97.9% and 88%, respectively, and the system’s ones to 97.5% and 83.9%, respectively. Not surprisingly, the accuracy was 96.9% and 95% for human and system observations, respectively.

The final test, to confirm the abovementioned results, was to compute a confusion matrix for detecting the accuracy of the AI system in detecting COVID+, pneumonia+, and healthy subjects. Our results suggest that the AI system reached a fascinating accuracy (93.8%), with a low misclassification rate (6.2%), with 93.8% weighted-F1.

Our results confirm that human evaluation was similar to that reported by Vicini et al. [22], showing 75% accuracy. On the other hand, AI evaluations of CXRs reported diagnostic values equal to or better than the current literature. Baltazar et al. [23] enrolled 335 healthy CXRs, 77 with COVID-19 pneumonia, and 565 CXRs with other than COVID-19 pneumonia. The authors, by using five different deep learning classification models (inceptionV3, Xception, MobileNet, VGG19, and InceptionResNetV2), reported a similar diagnostic value with 91–96% sensitivity, 94–98% specificity, and 90–96% PPV.

A study published by Rangarajan et al. [24], by enrolling 236 healthy CXRs, 47 classical and 138 indeterminate for COVID-19 pneumonia CXRs, reported that AI (the CheXnet deep neural network) showed 92% accuracy in classifying “normal” CXR into COVID or non-COVID. Moreover, Li et al. [25], by enrolling 154 CXRs with typical COVID-19 findings, aimed to determine the usefulness of AI-based methods (convolutional Siamese neural network) to implement diagnostic reliability among radiologists, reporting that the agreement can increase significantly when AI is applied to clinical practice.

Our results are similar to Salvatore et al. [26], who enrolled 98 CXRs of patients affected with COVID-19 pneumonia and 88 community-acquired pneumonia: the authors, by using an ensemble of deep neural networks (10 Resnet-50 trained on different subsets), reported an overall accuracy of 94% in the detection of lung pathological findings in comparison with healthy ones. Similarly, Castiglioni et al. [27], by training and validating an ensemble of ten ResNet-50 applied to two different centers, reported a maximum accuracy of 89%, testing 250 healthy CXRs and 250 with typical COVID-19 findings, in line with our presented results.

Zhang et al. [28], by enrolling in the final test 250 COVID-19 CXRs and 250 healthy CXRs by using an ensemble of 20 deep neural networks (DenseNet-121), reported an overall 92% accuracy in detecting lung involvement, corresponding to 88% sensitivity and 79% specificity, higher if compared with the 85% accuracy achieved by radiologists. Similarly, Wehbe, by evaluating more than 2985 COVID-19 CXRs, reported an overall accuracy of 88% after using an ensemble of six deep learning networks for image classification (Densenet-121, ResNet-50, Inception, Inception ResNet, Xception and EfficientNet-B2) [29]. Finally, Sun et al. [15], testing 2200 COVID-19 CXRs and 362,228 healthy CXRs, reported that the COVID-19 AI diagnostic system had worse accuracy (63.5% correct) than radiologist predictions.

A recent meta-analysis published by Islam et al. [30], evaluating nine studies with 2111 cases, reported that the CXR pooled sensitivity was 80.6% and its pooled specificity 71.5%, underlying that AI can have a pivotal role in increasing diagnostic accuracy, especially in clinical practice.

This study has some limitations. Our datasets comprised different numbers of CXRs across COVID-19 pneumonia and healthy subjects. We used an unbalanced dataset to evaluate typical bacterial pneumonia due to the main aim of our study, to test the usefulness of the AI system in identifying COVID-19 patients. To increase the robustness of our results, we decided to test each dataset singularly, firstly to avoid unbalanced effects.

Moreover, we tested the AI-based system on different cases from the same geographical area, which could have introduced a bias. Furthermore, we did not include clinical or laboratory data as complementary information for the diagnosis, even if these can add critical bias to the final radiological diagnosis. Finally, human readers and AI-based systems evaluated only CXRs acquired at the bedside, a typical approach during pandemics, especially in cases of severe disease when it is impossible to perform CXR in the orthogonal projections. In the future, we aim to test the system on the standard CXR to evaluate if the diagnostic accuracy can increase.

## 5. Conclusions

To conclude, our results show that this AI system could be helpful in everyday clinical practice to help radiologists detect COVID-19 pneumonia, especially in the emergency department where the number of patients and the quick response can play a pressing role in radiologists’ diagnosis. The possibility to highlight and distinguish the different patterns of lung infections via AI could aid in obtaining faster clinical decision making with a higher confidence level and improve the report of pulmonary diseases, shortening the turnaround time. Moreover, the AI system can also help radiologists detect common signs of typical bacterial pneumonia, being a crucial diagnostic resource in those countries where access to CT is lacking or in those cases with low diagnostic experience. We finally demonstrated that the application of the AI system can help classify patients into COVID+, pneumonia+, and healthy subjects with high accuracy and low misclassification rate, thus enhancing the diagnostic confidence of radiologists in everyday clinical practice, supporting the idea that the integration of AI in the reporting workstation, running in the background, can offer an easy way for obtaining a second opinion, increasing the accuracy with higher efficiency.

## Figures and Tables

**Table 1:** True positives (TPs), true negatives (TNs), false positives (FPs), and false negatives (FNs) for the radiologist and AI system.

		TPs (n, %)	TNs (n, %)	FPs (n, %)	FNs (n, %)
COVID-19+ vs. healthy (n = 2140)	Radiologist	1450 (67.8)	503 (23.5)	127 (5.9)	60 (2.8)
	AI	1419 (66.3)	515 (24.1)	127 (5.9)	79 (3.7)
Pneumonia+ vs. healthy (n = 1500)	Radiologist	1218 (81.2)	235 (15.6)	32 (2.2)	15 (1)
	AI	1191 (79.4)	234 (15.6)	45 (3)	30 (2)

**Table 2:** Diagnostic values for the radiologist and AI system.

		Sensitivity (%, 95% CIs)	Specificity (%, 95% CIs)	PPV (%, 95% CIs)	NPV (%, 95% CIs)	Accuracy (%, 95% CIs)
COVID-19+	Radiologist	96.0 (94.9–96.9)	79.8 (76.4–82.9)	91.9 (90.7–93.0)	89.3 (86.7–91.5)	91.2 (89.9–92.4)
	AI	94.7 (93.4–95.8)	80.2 (76.9–83.2)	91.7 (90.5–92.8)	86.7 (83.9–89.0)	90.3 (89.0–91.6)
Pneumonia+	Radiologist	97.9 (98.0–99.3)	88.0 (83.5–91.7)	97.4 (96.5–98.1)	94.0 (90.4–96.3)	96.9 (95.8–97.7)
	AI	97.5 (96.5–98.3)	83.9 (79.0–87.9)	96.3 (95.2–97.1)	88.6 (84.5–91.7)	95.0 (93.7–96.0)

## Data Availability

The data are unavailable due to privacy restrictions.
